# The Synthesis and Property Study of NH-Ac-Anchored Multilayer 3D Polymers

**DOI:** 10.3390/molecules30091981

**Published:** 2025-04-29

**Authors:** My Phan, Hao Liu, Lina M. Delgado, Hammed Olawale Faleke, Sai Zhang, Anthony F. Cozzolino, Dimitri Pappas, Guigen Li

**Affiliations:** 1 Department of Chemistry and Biochemistry, Texas Tech University, Lubbock, TX 79409-1061, USAlindelga@ttu.edu (L.M.D.);; 2 Continuous Flow Engineering Laboratory of National Petroleum and Chemical Industry, Changzhou University, Changzhou 213164, China

**Keywords:** multilayer 3D polymers, Suzuki–Miyaura cross-coupling, transition metal catalysis, aggregation-induced emission (AIE), gel permeation chromatography (GPC)

## Abstract

This study reports the synthesis, characterization, and property analysis of four novel multilayer 3D polymers (**1A** to **1D**) with 1,3-phenyl bridge architectures spanning 248 to 320 layers. High-molecular-weight polymers were successfully synthesized via catalytic Suzuki–Miyaura cross-coupling over a four-day reaction period. Structures, thermal, and optical properties were examined using multiple analytical techniques. Fourier transform-infrared (FT-IR) spectroscopy was used to study the hydrogen bonding within the polymer system, suggesting the formation of the polymer through the Suzuki–Miyaura coupling reaction. Ultraviolet–visible (UV-vis) spectroscopy indicated strong electronic delocalization, with maximum absorbance peaks between 257 and 280 nm. Thermal characterization, using differential scanning calorimetry (DSC) and thermogravimetric analysis (TGA), was used to investigate the thermal stability. TGA results showed that all four polymers retained more than 20% of their initial mass at 1000 °C, indicating good thermal stability across the series. DSC analysis revealed that polymer **1A** exhibited a glass transition temperature (Tg) of 167 °C, indicating the presence of a network formed by aromatic conjugation and hydrogen bonding. Furthermore, the subtle Tg step observed for **1A** suggests a degree of crystallinity within the polymer matrix, which was further supported by X-ray diffraction (XRD) analysis. Aggregation-induced emission (AIE) experiments provided further insights into intermolecular packing, and scanning electron microscopy (SEM) contributed to a better understanding of the morphology of the obtained polymers. These results highlight the potential of these polymers as thermally stable and conductive materials for biomedical and industrial applications.

## 1. Introduction

Polymers are among the most widely used materials, appearing in applications ranging from household products to advanced technological devices [1,2]. High-performance plastics are materials that surpass standard engineering plastics in terms of mechanical properties, chemical resistance, and/or heat stability. Among these, elevated thermal performance is often the defining characteristic. As a general guideline, a plastic is considered high-performance if it has short-term heat resistance of 250 °C and long-term heat resistance of 160 °C [3]. Despite their importance in industries like aerospace, automotive, and electronics, the high cost of high-performance plastics limits their use to specialized, low-volume applications [4,5,6]. The development of high-performance plastics relies on precise molecular design and advanced synthetic strategies. Typically, these polymers incorporate strong C=C bonds and aromatic units within their backbone structures to enhance thermal stability through resonance stabilization [7]. In addition, the Suzuki–Miyaura cross-coupling reaction is positioned as a cost-effective choice in organic synthesis, owing to the combination of affordable catalyst options, efficient processes (including operation under moderate temperature and pressure, as well as optimized energy use), and the availability of low-toxicity reagents [8,9].

A key strategy for designing such polymers is the Suzuki–Miyaura cross-coupling reaction, which enables the systematic construction of multilayer polymer architectures [10,11]. Previous designs predominantly utilized symmetrically or asymmetrically substituted aromatic rings at the para-position as bridge connectors between column anchors. In contrast, this study introduces a novel 1,3-substituted aromatic bridge architecture, which offers distinct structural and functional advantages. Specifically, derivatives of 1,3-bis(4,4,5,5-tetramethyl-1,3,2-dioxaborolan-2-yl)benzene and 1,8-dibromonaphthalene were employed as building blocks in an alternating fashion. The resulting polymers exhibit unique layered-column anchor arrangements and variable inter-anchor plane distances, diverging from previous frameworks. Notably, the naphthalene bridge heads align in non-parallel directions, introducing new structural variations that influence physical, chemical, and optical properties.

The incorporation of 1,3-substituted aromatic bridges could enhance solubility in tetrahydrofuran (THF) compared to 1,4-substituted counterparts, facilitating polymer characterization and expanding potential applications [10,12,13]. In particular, *N*-(3,5-dibromophenyl)acetamide was selected as a key component due to the unique properties of its acetamide functional group. This group serves as both a hydrogen bond donor and acceptor, enhancing solubility in polar solvents and promoting intra- and intermolecular hydrogen bonding, which can influence crystallinity and biological interactions [14,15,16]. Recent studies have shown that the acetamide group improves solubility and alters crystal packing through hydrogen bonding [17,18,19].

In this communication, we present our preliminary results on the synthesis of four new polymers and their structural, optical, and thermal properties, including electronic transitions, luminescence behavior, hydrogen bonding, thermal stability, glass transition characteristics, and morphology properties.

## 2. Results

**Scheme 1:**
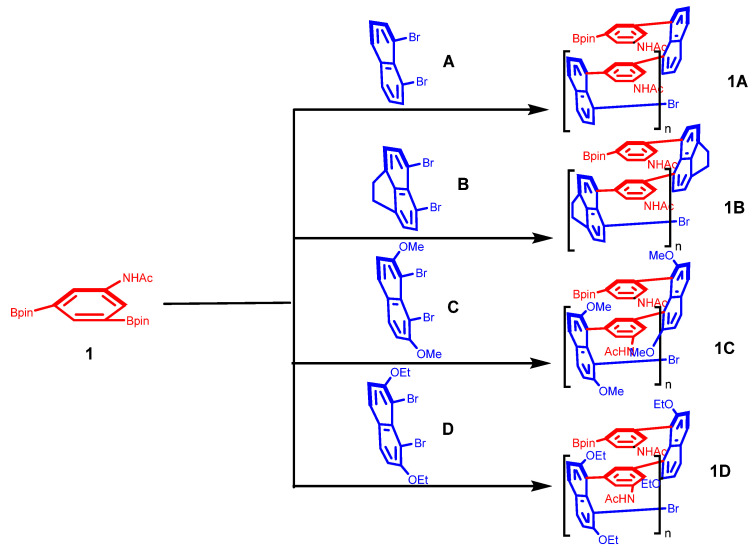
Multilayer polymers **1A**–**1D**. All reactions were carried out for combinations of co-monomer pairs of **1/A**, **1/B**, **1/C,** and **1D** on 0.2 mmol scale in the presence of Pd(S-BINAP)Cl_2_ (5 mol%) and KOAc in THF/H_2_O (5:1 mL) for 4 days under Ar protection.

As illustrated in Scheme 1, a total of four combinations of co-monomer pairs of **1/A**, **1/B**, **1/C**, and **1D** were subjected to modified Suzuki–Miyaura cross-coupling catalysis by using the first pair as a representative for detailed description. A molar ratio of 1:1 of 1,8-dibromonaphthalene 2 to *N*-(3,5-bis(4,4,5,5-tetramethyl-1,3-dioxolan-2-yl)phenyl)acetamide 1 was added to co-solvents of THF/H_2_O (5:1, *v*/*v*) in the presence of ((S)-2,2′-bis(diphenylphosphino)-1,1′-binaphthyl)dichloropalladium (Pd(S-BINAP)Cl_2_) (5% mol) as the catalyst alongside potassium acetate (KOAc) (4.0 equiv.) as an additive. Only very small amounts of polymeric products were formed for 24 h; the reaction mixture was then agitated at 88 °C for an extended duration of over four days until the consumption of the monomeric starting materials. The crude reaction products were subsequently purified by following standard procedures, yielding minimal small molecule impurities, as evidenced by the ^1^H-NMR analysis conducted in CHCl_3_-*d*_6_ solvent (illustrated in Figures S8–S11). The broad peaks observed across the entire spectrum indicate the presence of polymer signals. These chemical shifts found in the polymers are consistent with the expected values for the respective functional groups used in the polymerization reactions, confirming the successful incorporation of these groups into the polymer chains. The resulting solid was further subjected to drying processes to yield polymer **1A** as light-yellow solids with a yield of 74% (Scheme 1). The synthetic and analytical data pertaining to the four achiral polymers are summarized in Table 1. Gel permeation chromatography (GPC) was used to determine the molecular weights of polymers in the solution of THF by following the standard preparation of the sample solution. Although chiral catalyst (S)-2,2′-bis(diphenylphosphino)-1,1′-binaphthyl)dichloropalladium (Pd(S-BINAP)Cl_2_) was utilized for the present polymerization, we did not observe optical rotation of the resulting polymers **1A**–**1D**.

**Figure 1:**
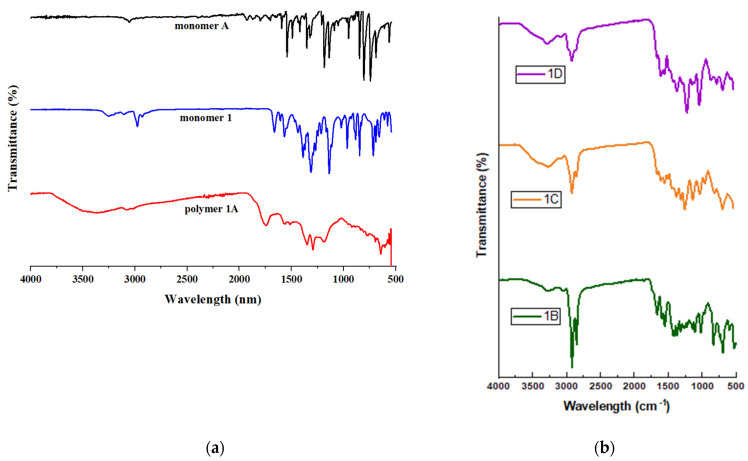
(**a**) FT-IR spectra of monomer 1, monomer 2, and polymer **1A**. (**b**) Comparative FT-IR spectra of polymers **1B**–**1D**.

To compare the structural characteristics of the individual monomers and their corresponding polymers, FT-IR spectroscopy was performed on monomer 1 (*N*-(3,5-bis(4,4,5,5-tetramethyl-1,3-dioxolan-2-yl)phenyl)acetamide), monomer 2 (1,8-dibromonaphthalene), and polymer **1A**. Figure 1a,b presents a comparison among the polymer series (**1A**–**1D**), allowing for a detailed analysis of their structural features and hydrogen-bonding behavior.

After normalizing the spectral curves (Figure 1), FT-IR analysis provided detailed insights into the polymer’s structural formation and hydrogen-bonding behavior. Hydrogen bonding induced notable changes in both the intensity and shape of the infrared bands. A prominent absorption band near 1600 cm⁻^1^—observed in polymers **1A**, **1B**, **1C**, and **1D**—was attributed to hydrogen-bonded carbonyl groups. These peaks were significantly broadened compared to those of free carbonyl groups, suggesting a distribution of hydrogen-bonding strengths within the samples [20]. In addition, the IR spectra revealed distinct structural transformations consistent with successful copolymer synthesis via the Suzuki–Miyaura coupling reaction. Compared to their respective monomers, the polymers exhibited broader bandwidths, supporting the formation of polyconjugated structures. One of the most pronounced spectral changes was observed in the –NH stretching region: whereas monomer 1 displayed only a minimal –NH stretching peak, polymers **1A**–**1D** showed a significantly broadened band. Aromatic –NH and C–H stretching frequencies were observed at approximately 3230 and 2933 cm⁻^1^, respectively, aligning well with the literature values for similar polyconjugated systems [21].

**Figure 2:**
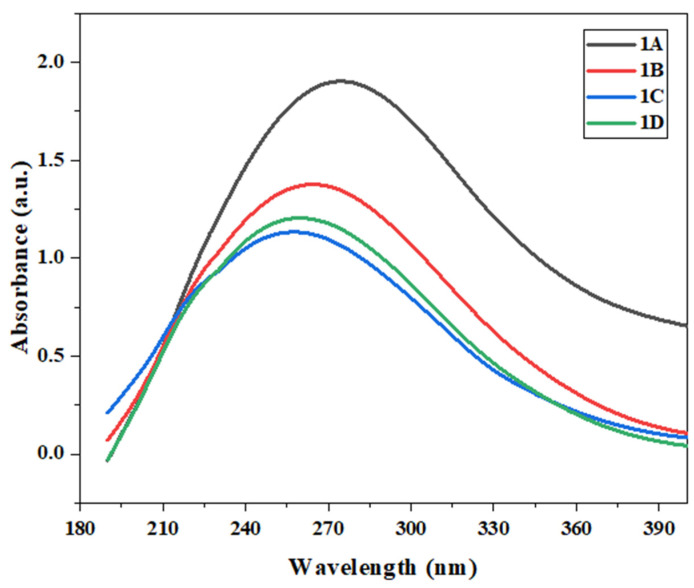
UV/vis absorption spectra of 01–04 in CHCl_3_; c = 0.05 mg mL^−1^.

UV-Vis spectroscopy provides insights into electron transitions, characterized by the promotion of electrons to higher energy states [22]. As shown in Figure 2, these polymers absorb light within the UV range, primarily due to the presence of conjugated π-bonding systems. The absorbance spectra of the polymers display notable similarities in their ultraviolet spectroscopic characteristics, which can be attributed to their closely related molecular structures. Only minor variations in alkyl chain configurations and band positions differentiate their spectra.

The highest absorbance peaks were observed within the wavelength range of 257–280 nm. The conjugated polymer, composed of alternating electron donor and acceptor groups, exhibits significant spectroscopic potential. This characteristic makes it suitable for applications such as creating donor–acceptor probes for fluorescent applications [23].

**Figure 3:**
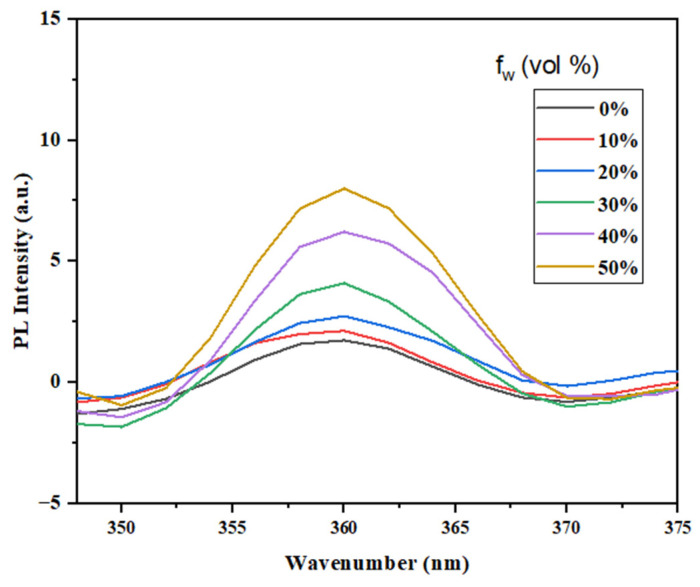
PL spectra of **1D** in THF/water mixtures with different water fractions (f_w_); c = 0.05 mg mL^−1^; excitation 324 nm; voltage 600 v.

Photoluminescence (PL) measurements were performed on all four polymers to evaluate their optical properties and aggregation behavior, as shown in Figure 3. Aggregation-induced emission (AIE) was observed in polymer **1D**, which can be attributed to the restriction of intramolecular rotation (RIR) mechanisms. The molecular architecture of polymer **1D** likely incorporates structural features that limit intramolecular rotational modes, effectively suppressing non-radiative decay pathways.

As illustrated in Figure 3, polymer **1D** exhibited a steady increase in emission maxima as the water fraction (f_w_) increased from 0% to 50%. This behavior suggests suppressed molecular motion due to intermolecular packing within the polymer matrix, a characteristic feature of AIE [24]. The unique multi-layered polymeric structure of **1D**, with parallel arrangements of intermolecular and intramolecular packing, results in more intricate molecular movements compared to those of small molecules or conventional non-layered polymers [10,12,13].

The strategic incorporation of steric hindrance elements appears to be a critical factor in activating the RIR process, enabling efficient radiative decay and enhanced light emission. In contrast, the other polymers did not exhibit significant AIE characteristics, underscoring the pivotal role of molecular design in modulating photophysical properties.

**Figure 4:**
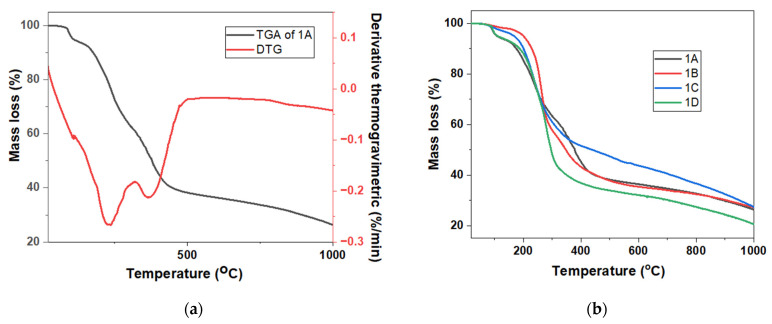
(**a**) TGA and derivative thermogravimetric (DTG) curves of polymer **1A**. (**b**) Comparative TGA curves of polymers **1A**–**1D**.

Thermal stability is a critical parameter in polymer material development, with thermogravimetric analysis (TGA) serving as a key technique to evaluate decomposition characteristics [25]. The weight loss versus temperature curves for polymers **1A**–**1D** are presented in Figure 4.

TGA analysis of the sample was performed from room temperature to 1000 °C. For polymers **1A** and **1D**, a distinct mass loss of approximately 6% was observed below 100 °C, likely due to the evaporation of residual solvents or absorbed moisture, with temperatures around 94–96 °C. This initial weight loss suggests the presence of physically trapped volatiles rather than any decomposition of the polymer backbone.

At 1000 °C, all four polymers retained more than 20% of their initial mass, indicating good thermal stability. The final residue percentages were approximately 26.5% for **1A**, 26.5% for **2A**, 27.6% for **3A**, and 20.7% for **4A**. The slightly higher char yield of polymer **3A** can be attributed to the presence of alkoxy (–OR) groups in its structure, which may lead to the formation of hydrogen bonding within the polymer system. This appears to play a significant role in enhancing thermal stability. Hydrogen bonds can create a more stabilized, interconnected network, reducing the extent of volatilization and contributing to higher residual mass after heating. This interpretation is further supported by the IR spectra, where polymers **3A** and **4A** exhibited a more broadened absorption peak near 3400 cm⁻^1^ compared to **1A** and **2A**—indicative of stronger or more extensive hydrogen bonding. Despite having a similar final residue to **1A**, polymer **2A** features a longer alkyl side chain, which may reduce overall thermal stability. As a result, **2A** showed the lowest final residue percentage among the four, likely due to the increased flexibility and reduced packing efficiency associated with longer alkyl chains [26].

Polymer degradation primarily happens through the breaking of the main chains or side chains of macromolecules. In nature, this process is triggered by factors such as thermal activation, hydrolysis, biological activity (e.g., enzymes), oxidation, photolysis, or radiolysis [27]. Thermal degradation typically results in weight loss in O_2_ or N_2_ environments, which can be measured using TGA, a technique that has long been regarded as a reliable method for studying the thermal stability and degradation of polymers [28]. Thermogravimetric curves reveal the decomposition mechanism of polymers, helping to define their characteristics and aid in identification. Polymer degradation can be classified into the following mechanisms: main-chain scission, side-group scission, elimination, depolymerization, cyclization, and cross-linking [29]. The susceptibility of a polymer to degradation is influenced by its structure. Epoxies and polymers with aromatic functionality are particularly prone to ultraviolet degradation, while hydrocarbon-based polymers are more vulnerable to thermal degradation and are generally not suited for high-temperature applications [30].

The observed thermal stability can be attributed to the polymer’s unique molecular structure, characterized by highly stable chemical bonds. Bond strength, quantified by bond dissociation energy, plays a critical role in thermal resistance. For instance, carbon–carbon double bonds (C=C) require higher energy to break (123 kcal/mol) compared to single bonds (C–C, 83 kcal/mol). High-performance plastics often incorporate C=C bonds in their molecular backbones to enhance stability. Furthermore, the strategic inclusion of aromatic units contributes to thermal stability through resonance stabilization [9].

In TGA analysis, the final residue provides a useful measure of thermal stability. For instance, common polymers such as polystyrene (PS) decompose almost completely at 1000 °C, leaving virtually no residue, while polyethylene (PE) and polypropylene (PP) retain only about 0.44 wt% at the same temperature [31,32].

**Figure 5:**
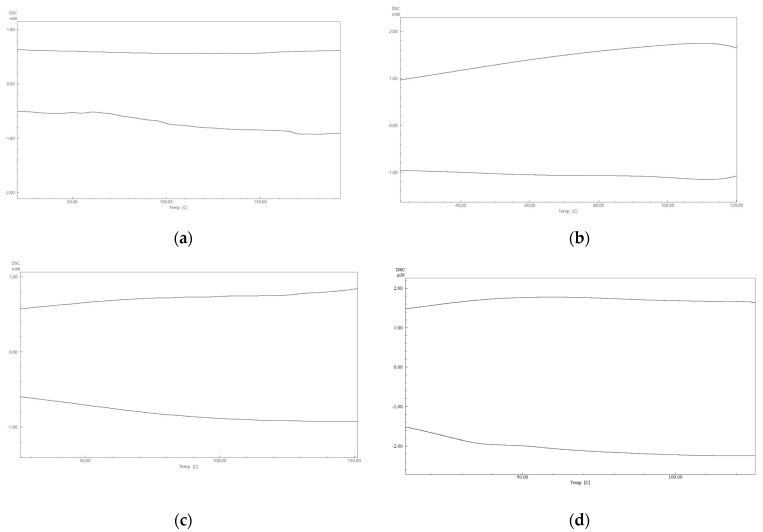
DSC thermograms of four polymers (**a**) **1A**, (**b**) **1B**, (**c**) **1C**, and (**d**) **1D**.

DSC thermograms of the four polymers **1A**–**1D** are shown below, as illustrated in Figure 5. All four polymers exhibited weak or broad transitions in their DSC curves, a behavior commonly reported in highly cross-linked, crystalline, or biopolymer systems [33,34,35,36,37].

**Figure 6:**
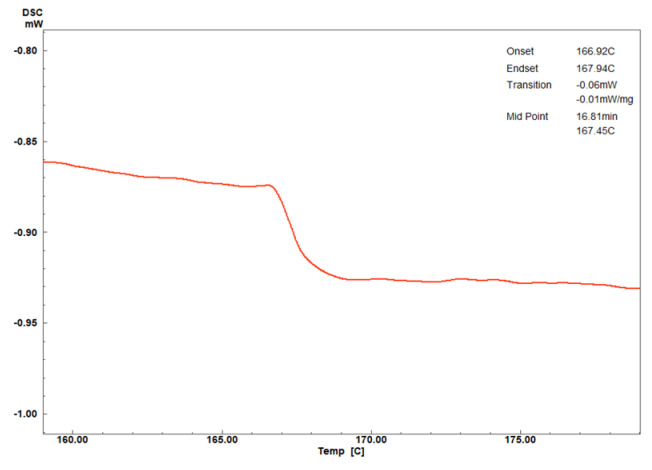
Zoom-in of DSC curves as a function of temperature (°C) from Figure 1a.

No clear melting or crystallization transitions (Tm or Tc) were observed within the measured temperature range. This absence may be attributed to the polymer’s highly rigid backbone and strong intra- and intermolecular hydrogen bonding [38,39]. The glass transition temperature (Tg) of polymer **1A** was identified at approximately 167 °C during the third heating cycle (Figure 6). This relatively high Tg reflects the dense molecular network stabilized by extensive hydrogen bonding. Tg marks the transition from a glassy, rigid state—exhibiting high strength and hardness—to a softer, rubbery state with reduced structural integrity at elevated temperatures [38,39]. A subtle step change near Tg was also observed, which may be linked to the polymer’s degree of crystallinity. This is further supported by the X-ray diffraction (XRD) results, which show strong diffraction patterns indicative of high crystallinity. In general, high crystallinity can diminish the prominence of the Tg step, as crystalline regions restrict the mobility of amorphous chains by acting as physical cross-links [38,39].

**Figure 7:**
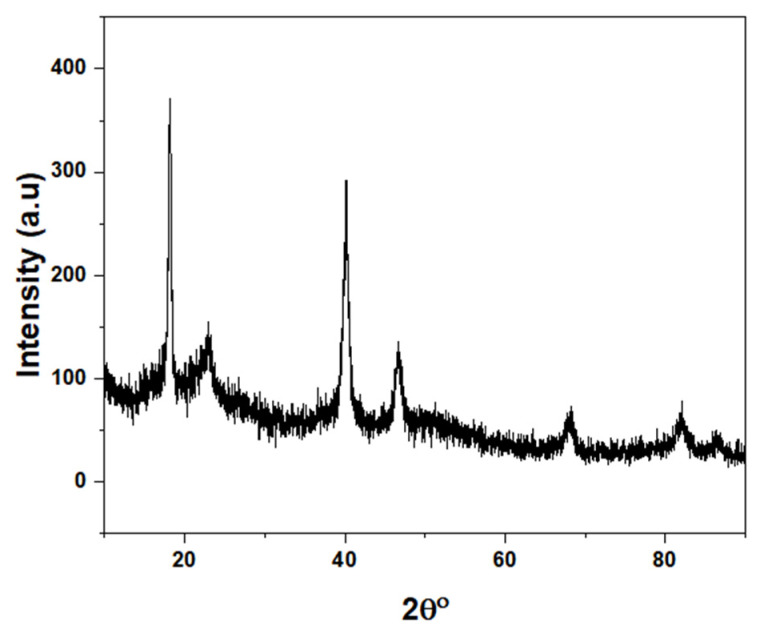
XRD spectra of polymer **1A**.

XRD measurements were carried out using a Rigaku MiniFlex II powder diffractometer with Cu-Kα radiation (λ = 0.154 nm) in Bragg–Brentano geometry. Diffraction patterns were recorded over a 2θ range of 3° to 90° with a step interval of 0.02°. The XRD data (Figure 7) reveals the highly crystalline nature of the polymer. The presence of sharp, well-defined peaks at around 20° and 40° indicates a high degree of long-range order within the material.

**Figure 8:**
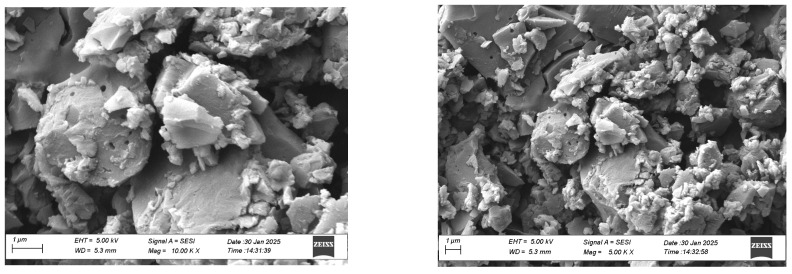
SEM results for polymer **1A** at different magnifications (10.00 K× (**left**) and 5.00 K× (**right**)).

Morphological analysis of polymer **1A** was conducted using scanning electron microscopy (SEM) at different magnifications. The polymer samples were coated with a thin gold layer to improve conductivity and minimize signal-to-noise interference during the data acquisition. As illustrated in Figure 8, the SEM images of polymer **1A** revealed a surface characterized by irregularly distributed particles with dimensions ranging from approximately 2 to 10 μm. Polymer **1A** exhibited a rough texture with a granular morphology, lacking distinct uniformity.

## 3. Materials and Methods

All experimental protocols were carried out using magnetic stirring in glassware that had been completely dried in an oven, utilizing anhydrous solvents in an inert argon atmosphere. Syringes, stainless steel or polyethylene cannulas, rubber septa, and a gentle counter-flow of argon were employed for the introduction of solvents, liquids, and solutions. Cooling baths made of ice/water (0 °C) or dry ice/acetone (−78 °C) were set up in Dewar vessels. Elevated temperature reactions were performed using heated oil baths. The removal of solvents was accomplished through rotary evaporators working at temperatures between 40 and 65 °C. The yields presented in this report are categorized as distinct chromatographic and nuclear magnetic resonance (NMR) yields. All commercially sourced chemicals were utilized as received, with no further purification. Solvents like methanol (CH_3_OH), toluene, ethyl acetate (EA), ether, dichloromethane (DCM), dioxane, and acetone were used directly without additional purification. An advanced solvent delivery system from Innovation Technology supplies both tetrahydrofuran (THF) and DCM. The ^1^H and ^13^C NMR spectra were obtained on 400 MHz and 500 MHz spectrometers using tetramethylsilane (TMS) as the internal standard. The residual solvent signal (δ = 7.26 for CDCl_3_) served as a reference for the ^1^H NMR spectra. Chemical shifts (δ) relative to TMS were reported in parts per million (ppm). The data were defined by chemical shifts, multiplicity (singlet, doublet, triplet, multiplet), coupling constants (J, Hz), and integration values. The TOSOH EcoSEC HLC-8420 gel permeation chromatography (GPC) system, equipped with a dual-flow refractive index detector, was used for GPC data collection (Tosoh Bioscience Inc., South San Francisco, CA, USA). In addition to the refractive index detector, a UV detector was also included for the assessment of UV-visible polymers. The operational range of the installed columns extends from 500 to 107 Daltons. Sample analyses were performed at a flow rate of 0.7 mL/min over a period of 25 min. For calibration purposes, polystyrene (PS) standards were used in our studies. Infrared (IR) spectra were obtained using a PerkinElmer Spectrum Two (PerkinElmer, Seer Green, UK) Fourier-transform infrared (FT-IR) spectrometer (wavenumbers expressed in cm^−1^) along with an FT-IR spectrometer Nicolet iS20 (Thermo Scientific, Madison, WI, USA) that is equipped with an attenuated total reflectance (ATR) accessory. Thermogravimetric analysis (TGA) was performed on a 5.8 mg sample utilizing an alumina pan within a nitrogen atmosphere, maintained at a constant gas flow rate of 100 mL/min, employing a Mettler Toledo TGA/SDTA851e module (Shimadzu Scientific Instrument, Inc., Columbia, MA, USA). Differential scanning calorimetry (DSC) was executed with a 10 mg sample under identical nitrogen atmosphere conditions, with a reduced flow rate of 50 mL/min, using an aluminum pan and a Mettler Toledo DSC822e module (Shimadzu Scientific Instrument, Inc., Columbia, MA, USA). Analytical measurements were processed and analyzed using LabSolutionsTA (version 1.01) and OriginPro 2021 (version 9.8.0.200).

## 4. Conclusions

In this study, four multilayer 3D polymers (**1A** to **1D**) were designed and synthesized, with theoretical layer counts ranging from 248 to 320, utilizing 1,3-position aromatic bridge architectures. Comprehensive characterization was performed using multiple analytical techniques to investigate their structural, optical, and thermal properties. FT-IR spectroscopy was used to investigate the hydrogen bonding within the polymer system, suggesting polymer formation via the Suzuki–Miyaura coupling reaction and revealing aromatic backbone structures. This structural configuration suggests the potential for a cross-linked network, which could enhance the material’s structural integrity and thermal stability. Further thermal analysis was conducted using TGA and DSC. While the TGA results showed that all four polymers retained more than 20% of their initial mass at 1000 °C, indicating good thermal stability, complementary DSC analysis identified a glass transition temperature (Tg) for polymer **1A** at approximately 167 °C. This Tg, along with a subtle step transition, suggests the presence of a polymer network formed through aromatic conjugation and hydrogen bonding, as well as a degree of crystallinity within the polymer matrix, as confirmed by X-ray diffraction (XRD) analysis. Overall, these results point to the material’s good thermal stability and its potential suitability for high-temperature applications in advanced industrial and biomedical settings. In addition to thermal characterization, UV-Vis spectroscopy and AIE (aggregation-induced emission) experiments provided insights into the optical properties and aggregation behavior of these polymers. Notably, polymer **1D** exhibited the AIE phenomenon, attributed to intermolecular packing within the polymer matrix, a characteristic of its unique structural design. In addition, the SEM analysis is used to investigate the morphology of the obtained polymers. The structural, thermal, and optical characteristics of these multilayer 3D polymers position them as promising candidates for advanced materials research and a broad range of technological applications.

## Figures and Tables

**Table 1:** Results of synthetic polymers.

Poly-Product	Yield ^a^ (%)	Mn ^b^	Mw ^b^	PDI ^c^	Theor. Layers ^d^
**1A**	62	68,016	82,583	1.214	320
**1B**	34	72,803	84,381	1.159	330
**1C**	70	65,917	80,674	1.224	254
**1D**	50	69,165	85,674	1.239	248

^a^ Isolated yield based on a co-monomer for each case (co-monomer ratio = 1:1). ^b^ Determined by GPC with a polystyrene standard. ^c^ PDI = Mw/Mn. ^d^ Based on Mn of GPC determination.

## Data Availability

All data are available in the manuscript or Supplementary Materials.

## Supplementary Materials

The following supporting information can be downloaded at: https://www.mdpi.com/article/10.3390/molecules30091981/s1, Figure S1. ^1^H NMR of compound **2**. Figure S2. ^1^H NMR of compound **1**. Figure S3. ^1^H NMR of compound 3. Figure S4. ^1^H NMR of compound **B**. Figure S5. ^1^H NMR of compound **4**. Figure S6. ^1^H NMR of compound **C**. Figure S7. ^1^H NMR of compound **D**. Figure S8. ^1^H NMR spectrum of **1A**. Figure S9. ^1^H NMR spectrum of **1B**. Figure S10. ^1^H NMR spectrum of **1C**. Figure S11. ^1^H NMR spectrum of **1D**. Figure S12. GPC data of **1A**. The inset shows the zoomed in rectangular area. Figure S13. GPC data of **1B**. The inset shows the zoomed in rectangular area. Figure S14. GPC data of **1C**. The inset shows the zoomed in rectangular area. Figure S15. GPC data of **1D**. The inset shows the zoomed in rectangular area [40,41,42].
