# Supplementary material for: The Synthesis and Property Study of NH-Ac-Anchored Multilayer 3D Polymers
Source: Molecules. 2025 Apr 29;30(9):1981. doi: 10.3390/molecules30091981 (PMC12073231; doi:10.3390/molecules30091981)
Supplement: Supplementary file 1 [file molecules-30-01981-s001.zip › molecules-3514105-supplementary.pdf]

The Synthesis and Property Study of NH-Ac-Anchored Multilayer 3D Polymers

My Phan,[1] Hao Liu,[1] Lina M. Delgado,[1] Hammed Olawale Faleke,[1] Sai Zhang,[2] Anthony F. Cozzolino,[1] Dimitri Pappas,[1] and Guigen Li[1,*]

[1] *Department of Chemistry and Biochemistry, Texas Tech University, Lubbock, Texas 79409-1061, USA.*

[2] *Continuous Flow Engineering Laboratory of National Petroleum and Chemical Industry, Changzhou University, Changzhou, Jiangsu 213164, China.*

[*]*Correspondence should be addressed to Guigen Li: guigen.li@ttu.edu (GL)*

Materials and Methods

S1.1. Materials and Measurements

All experimental protocols were carried out using magnetic stirring in glassware that had been completely dried in an oven, utilizing anhydrous solvents in an inert argon atmosphere. Syringes, stainless steel or polyethylene cannulas, rubber septa, and a gentle counter-flow of argon were employed for the introduction of solvents, liquids, and solutions. Cooling baths made of ice/water (0 °C) or dry ice/acetone (-78 °C) were set up in Dewar vessels. Elevated temperature reactions were performed using heated oil baths. The removal of solvents was accomplished through rotary evaporators working at temperatures between 40 to 65 °C. The yields presented in this report are categorized as distinct chromatographic and nuclear magnetic resonance (NMR) yields. All commercially sourced chemicals were utilized as received, with no further purification. Solvents like methanol (CH_3OH), toluene, ethyl acetate (EA), ether, dichloromethane (DCM), dioxane, and acetone were used directly without additional purification. An advanced solvent delivery system from Innovation Technology supplies both tetrahydrofuran (THF) and DCM. The ^1H and ^{13}C NMR spectra were obtained on 400 MHz and 500 MHz spectrometers using tetramethylsilane (TMS) as the internal standard. The residual solvent signal (δ = 7.26 for $CDCl_3$) served as a reference for the ^1H NMR spectra. Chemical shifts (δ) relative to TMS were reported in parts per million (ppm). The data were defined by chemical shifts, multiplicity (singlet, doublet, triplet, multiplet), coupling constants (J, Hz), and integration values. The TOSOH EcoSEC HLC-8420 gel permeation chromatography (GPC) system, equipped with a dual-flow refractive index detector, was used for GPC data collection. In addition to the refractive index detector, a UV detector is also included for the assessment of UV-visible polymers. The operational range of the installed columns extends from 500 to 107 Daltons. Sample analyses were performed at a flow rate of 0.7 mL/min over a period of 25 minutes. For calibration purposes, polystyrene (PS) standards were used in our studies. Infrared (IR) spectra were obtained using a PerkinElmer Spectrum Two (PerkinElmer, Seer Green, UK) Fourier-transform infrared (FT-IR) spectrometer (wavenumbers expressed in cm−1) along with an FT-IR spectrometer Nicolet iS20 (Thermo Scientific, Madison, USA) that is equipped with an attenuated total reflectance (ATR) accessory. Thermogravimetric analysis (TGA) was performed utilizing an alumina pan within a nitrogen atmosphere, maintained at a constant gas flow rate of 100 mL/min, employing a DTG-60H from Shimadzu. Differential scanning calorimetry (DSC) was executed under identical nitrogen atmosphere conditions, with a reduced flow rate of 50 mL/min, using an aluminum pan and a DSC-60 Plus from Sihmadzu. Analytical measurements were processed and analyzed using TA-TRIOS software and Origin software. The polymer samples were sputter-coated with a thin gold layer, and the morphological characteristics were examined using a NANOSCIENCE Phenom ProX scanning electron microscope (SEM) (Phoenix, AZ, USA). Single-crystal X-ray diffraction (SCXRD) data were collected using a Rigaku Synergy-S diffractometer equipped with both Cu-Kα and Mo-Kα radiation sources and a HyPix-6000HE hybrid photon counting detector. The instrument allows for precise molecular structure determination across a wide temperature range (100–400 K) using an Oxford Cryostream 700. Data integration was performed using CrysAlisPro, and structure solution and refinement were carried out with SHELXT (https://journals.iucr.org/a/issues/2015/01/00/sc5086/index.html) and SHELXL (https://journals.iucr.org/c/issues/2015/01/00/fa3356/index.html) respectively utilizing the Olex2 GUI. XRD measurements were carried out using a Rigaku MiniFlex II powder diffractometer equipped with a D/teX Ultra 1D silicon strip detector and operated in Bragg-

Brentano geometry with Cu-Kα radiation (λ = 0.154 nm). Diffraction patterns were recorded over a 2θ range of 3° to 90° with a step interval of 0.02°.

S1.2. Synthetic Procedure

Synthesis of N-(3,5-dibromophenyl)acetamide (2)

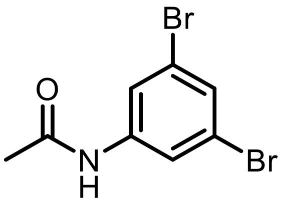

After the dissolution of 3,5-dibromoaniline (1.0 g, 4.53 mmol) in approximately 20 mL of CH_2Cl_2, acetic anhydride (0.45 g, 4.41 mmol) alongside K_2CO_3 (0.63 g, 4.56 mmol) was subsequently introduced and thoroughly mixed at ambient conditions. Following the completion of the reaction, the resultant mixture underwent extraction utilizing CH_2Cl_2 and water. The organic phase was then subjected to drying with $MgSO_4$ and concentrated to enhance purity. The purification procedure employed a silica gel column with hexane and dichloromethane as eluting solvents, resulting in the acquisition of a white solid (82.15% yield). ^{1}H NMR (400 MHz, CHLOROFORM-D) δ 7.65 (s, 2H), 7.39 (s, 1H), 7.09 (s, 1H), 2.17 (s, 3H).

Synthesis of N-[3,5-Bis(4,4,5,5-tetramethyl-1,3,2-dioxaborolan-2-yl)phenyl]acetamide (1)

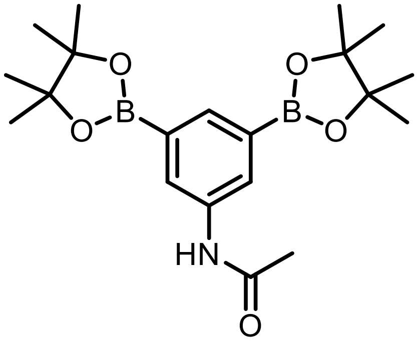

A solution comprising N-(3,5-dibromophenyl)acetamide (515 mg, 1.76 mmol, 1 equivalent) and bis(pinacolato)diboron (900 mg, 3.53 mmol, 2 equivalents) was prepared in 20.0 mL of anhydrous and degassed DMF. Then, Pd(dppf)Cl_2 (130 mg, 0.17 mmol, 10 mol%) and KOAc (1.04 g, 10.60 mmol, 6 equivalents) were introduced into the reaction mixture. The reaction mixture was subjected to heating at a temperature of 80°C for a duration of 24 hours. Upon completion of the reaction, the solvents were removed through evaporation, and the resultant crude product was purified via column chromatography using silica gel with hexane and ethyl acetate as eluting solvents to yield the final product (78% yield). ^{1}H NMR (400 MHz, CHLOROFORM-*D*) δ 7.98 (s, 2H), 7.97 (s, 1H), 7.71 (s, 1H), 2.13 (s, 3H), 1.28 (s, 24H).

Synthesis of 2,7-dimethoxynaphthalene (3)

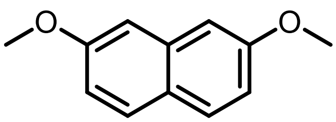

A solution of 2,7-dihydroxynaphthalene (5.0 g, 31.25 mmol) in acetone (156 mL) was treated with potassium carbonate (K_2CO_3) (21.5 g, 125 mmol). Iodomethane (11.6 mL, 187.5 mmol) was added, and the resulting mixture was stirred under reflux for 18 hours. Once the reaction concluded, the mixture was allowed to cool to ambient temperature. The reaction mixture was then concentrated, and the residue was extracted with EtOAc (3 x 50 mL). The combined organic extracts underwent washing with water (2 x 50 mL) and brine (2 x 50 mL). The solution was dried using Na_2SO_4 and then concentrated under reduced pressure. Ultimately, the product was recrystallized from methanol, resulting in the desired compound as a white solid with the yield of 69% (4.03 g). The spectral data corresponded with that which has been previously documented in the academic literature.[1] ^{1}H NMR (400 MHz, CHLOROFORM-*D*) δ 7.66 (dt, *J* = 9.1, 0.6 Hz, 2H), 7.06 (dd, *J* = 2.4, 0.7 Hz, 2H), 7.01 (d, *J* = 2.5 Hz, 1H), 6.99 (d, *J* = 2.5 Hz, 1H), 3.91 (s, 6H).

Synthesis of 1,8-dibromo-2,7-dimethoxynaphthalene (B)

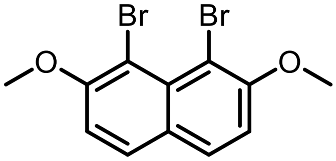

N-Bromosuccinimide (NBS) (8.16 g, 45.8 mmol) along with pyridine (2.5 mL, 2.4 g, 30 mmol) were dissolved in 70 mL of chloroform. The resulting mixture underwent refluxing under a nitrogen atmosphere for a period of 1 hour. Afterward, a solution of 2,7-dimethoxynaphthalene (2.16 g, 11.5 mmol) in 12.5 mL of chloroform was added, and the reflux was continued for an additional 9 hours. Once this step was finished, the mixture was allowed to cool to room temperature before being promptly adsorbed onto silica gel. The flash chromatography process, using hexane and ethyl acetate as eluting solvents, produced B as a pale yellow solid (85%). The spectral data corresponded with that which has been previously documented in the academic literature.[2] ^{1}H NMR (400 MHz, CHLOROFORM-*D*) δ 7.75 – 7.71 (m, 1H), 7.15 (d, *J* = 9.0 Hz, 1H), 4.01 (s, 3H).

Synthesis of 2,7-diethoxynaphthalene (4)

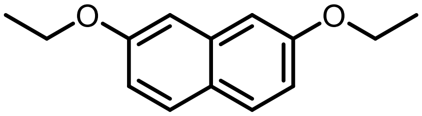

A solution of 2,7-dihydroxynaphthalene (5.0 g, 31.25 mmol) in DMF (156 mL) was treated with K_2CO_3 (21.5 g, 125 mmol). Ethyl bromide (13.9 mL, 187.5 mmol) was added, and the resulting mixture was stirred under reflux for 18 hours. Once the reaction concluded, the mixture was allowed to cool to ambient temperature. The reaction mixture was then concentrated, and the residue was extracted with EtOAc (3 x 50 mL). The combined organic extracts underwent washing with water (2 x 50 mL) and brine (2 x 50 mL). The solution was dried using Na_2SO_4 and then concentrated under reduced pressure. Ultimately, the product was recrystallized from methanol, resulting in the desired compound as a white solid with the yield of 60%. The spectral data corresponded with that which has been previously documented in the academic literature.[1] ^{1}H NMR (400 MHz, CHLOROFORM-*D*) δ 7.64 (dt, *J* = 9.0, 0.6 Hz, 1H), 7.04 – 6.95 (m, 2H), 4.13 (q, *J* = 7.0 Hz, 2H), 1.47 (t, *J* = 7.0 Hz, 3H).

Synthesis of 1,8-dibromo-2,7-diethoxynaphthalene (C)

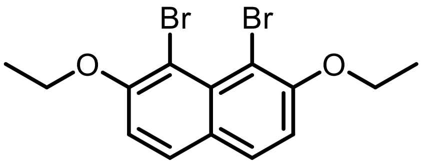

NBS (8.16 g, 45.8 mmol) along with pyridine (2.5 mL, 2.4 g, 30 mmol) were dissolved in 70 mL of chloroform. The resulting mixture underwent refluxing under a nitrogen atmosphere for a period of 1 hour. Afterward, a solution of 2,7-diethoxynaphthalene (2.16 g, 11.5 mmol) in 12.5 mL of chloroform was added, and the reflux was continued for an additional 9 hours. Once this step was finished, the mixture was allowed to cool to room temperature before being promptly adsorbed onto silica gel. The flash chromatography process, using hexane and ethyl acetate as eluting solvents, produced C as a pale yellow solid (80%). The spectral data corresponded with that which has been previously documented in the academic literature.[2] ^{1}H NMR (400 MHz, CHLOROFORM-*D*) δ 7.65 (d, *J* = 9.0 Hz, 1H), 7.08 (d, *J* = 8.9 Hz, 1H), 4.19 (q, *J* = 7.0 Hz, 2H), 1.49 (t, *J* = 7.0 Hz, 3H).

Synthesis of 5,6-dibromoacenaphthene (D)

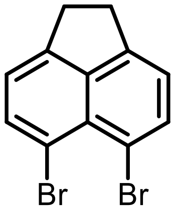

A solution of NBS in DMF (38.91 mmol) was introduced into a chilled mixture of acenaphthene (1) in DMF (12.97 mmol). The reaction mixture was stirred for 3 hours at temperatures between 0-5 ˚C and subsequently for 12 hours at 10-15 ˚C. The resulting precipitate was filtered, rinsed with ethanol, and dried under vacuum to yield a white powder of 5, 6-dibromo acenaphthene with a yield of 35%.[3] ^{1}H NMR (400 MHz, CHLOROFORM-*D*) δ 7.77 (dtd, *J* = 16.7, 8.3, 4.7 Hz, 1H), 7.08 (dt, *J* = 15.9, 8.1 Hz, 1H), 3.41 – 3.19 (m, 2H).

Reference

1. Jolliffe, J.D.; Armstrong, R.J.; Smith, M.D. Catalytic Enantioselective Synthesis of Atropisomeric Biaryls by a Cation-Directed O-Alkylation. *Nat. Chem.* **2017**, *9*, 558–562, doi:10.1038/nchem.2710.
2. Kuwano, R.; Morioka, R.; Kashiwabara, M.; Kameyama, N. Catalytic Asymmetric Hydrogenation of Naphthalenes. *Angew. Chem. Int. Ed Engl.* **2012**, *51*, 4136–4139, doi:10.1002/anie.201201153.
3. Vijay, V.; Ramakrishnan, R.; Hariharan, M. Halogen–Halogen Bonded Donor-Acceptor Stacks Foster Orthogonal Electron and Hole Transport. *Cryst. Growth Des.* **2021**, *21*, 200–206, doi:10.1021/acs.cgd.0c01024.

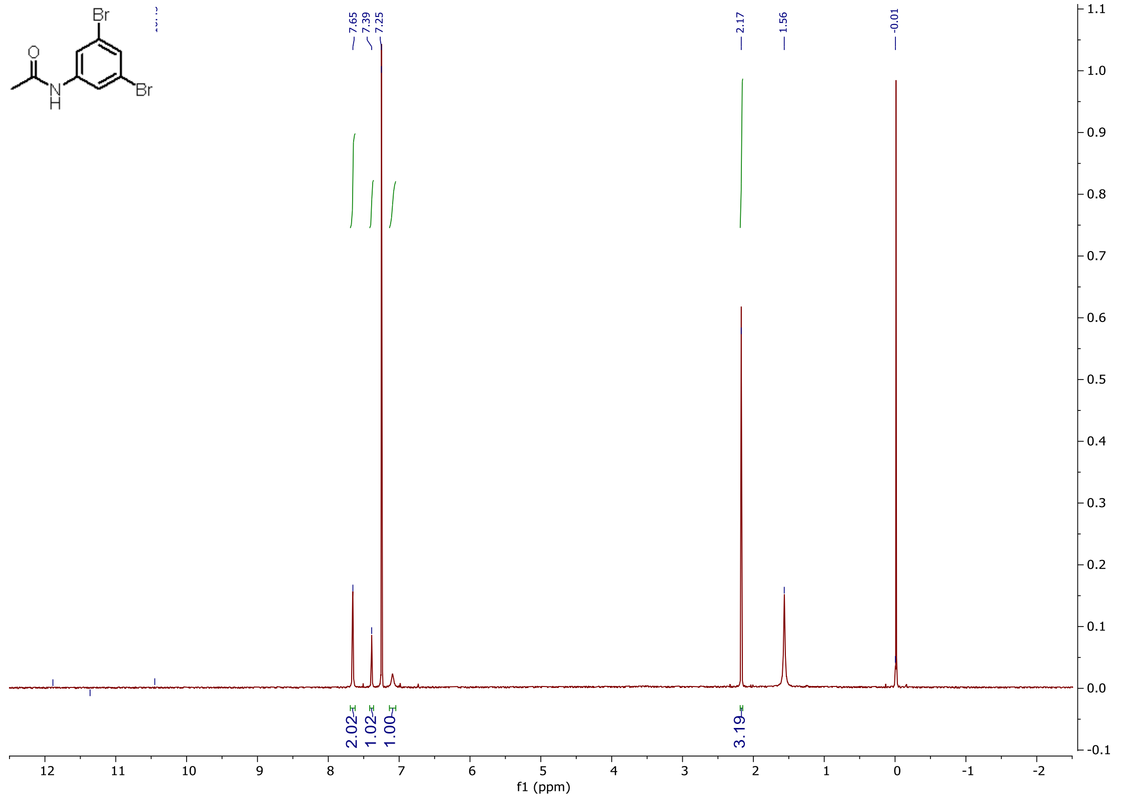

Figure S1. ^{1}H NMR of compound 2

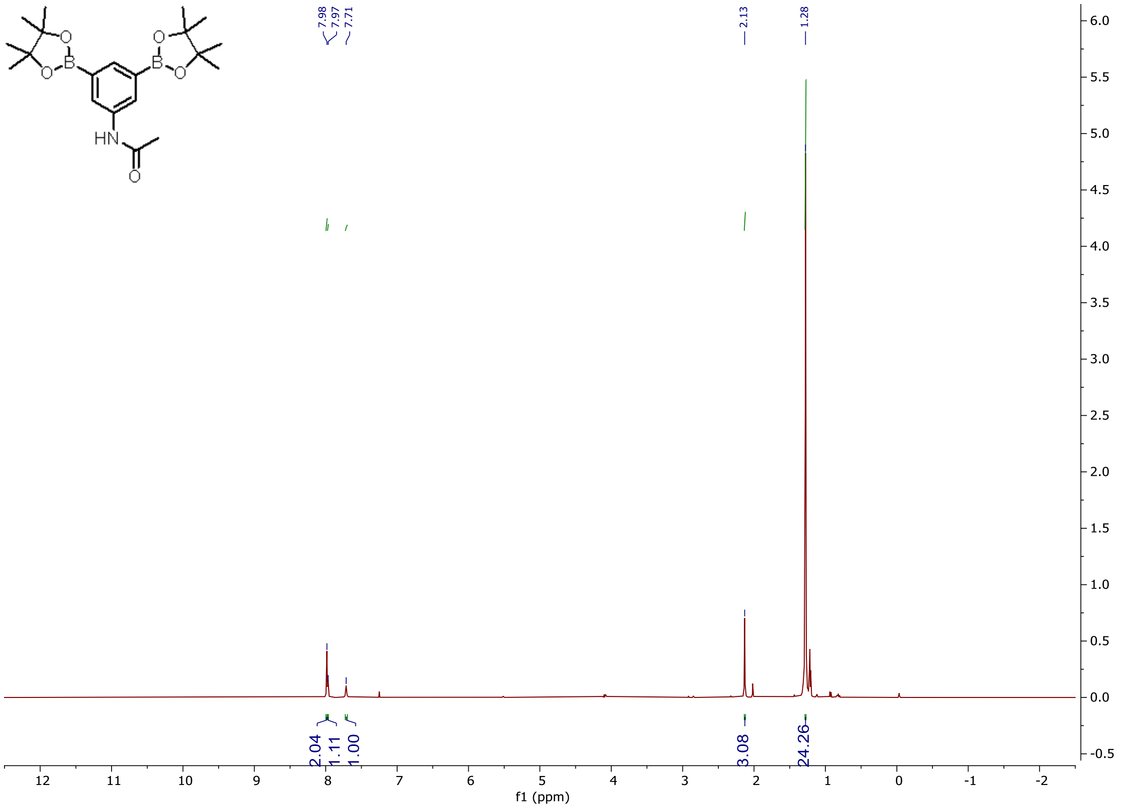

Figure S2. ^{1}H NMR of compound 1

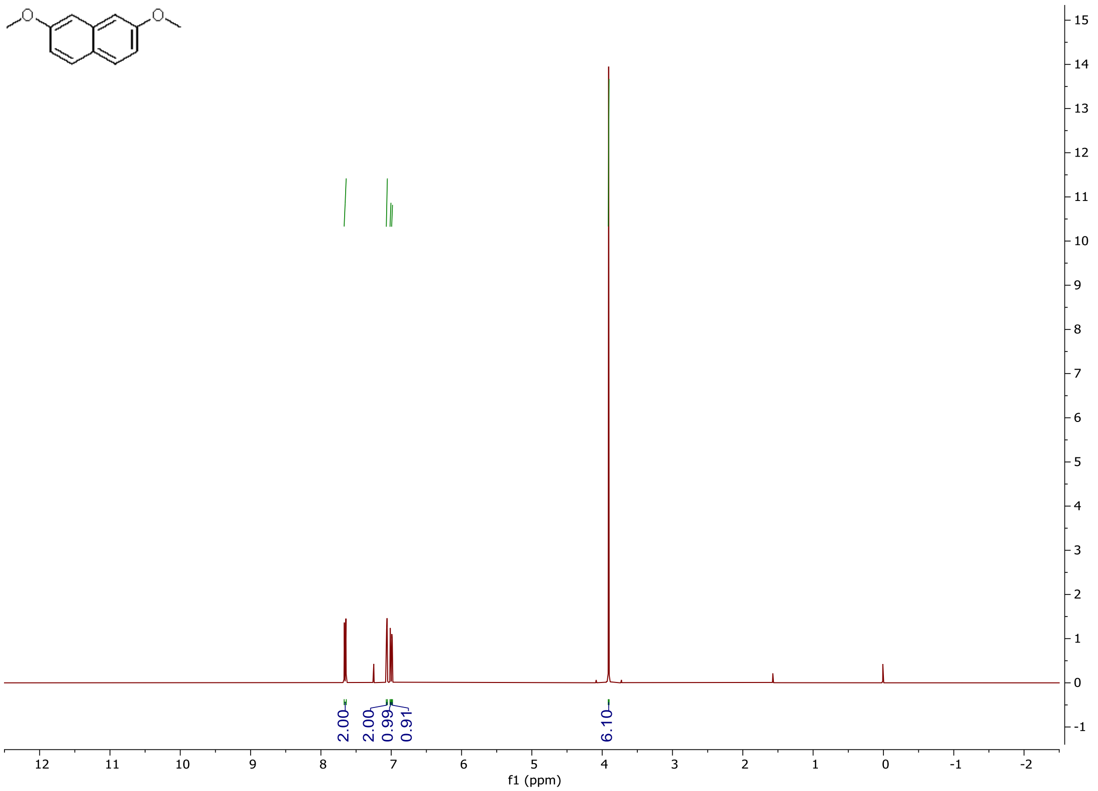

Figure S3. ^{1}H NMR of compound 3

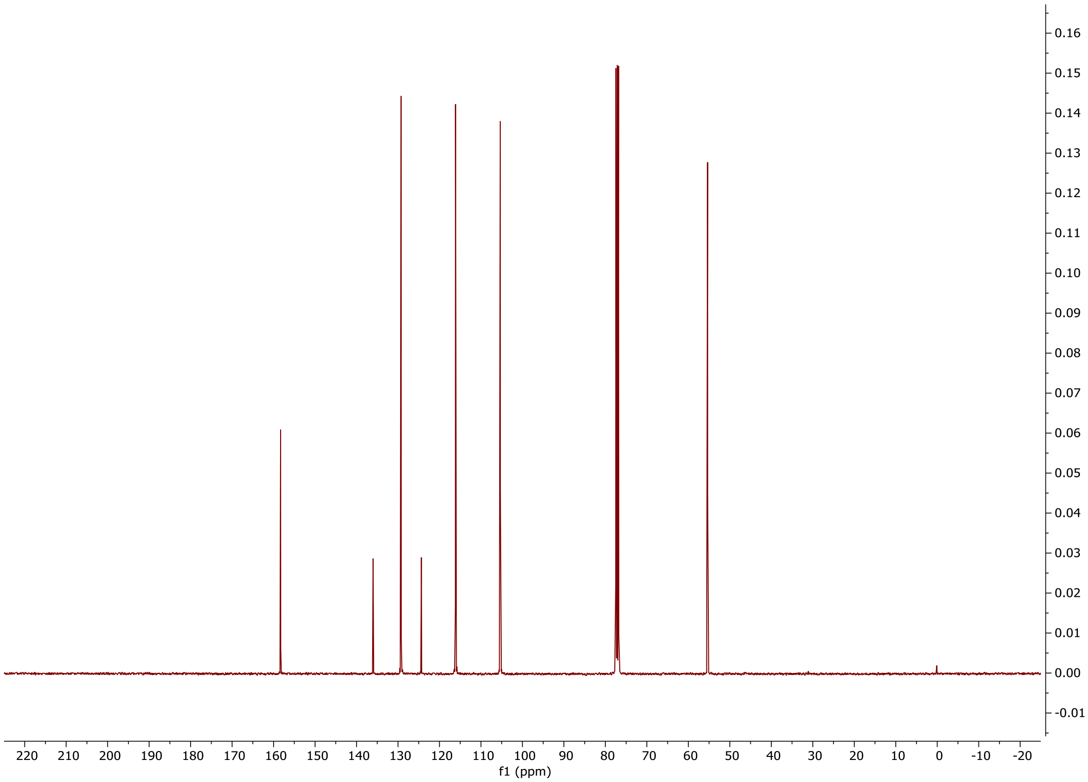

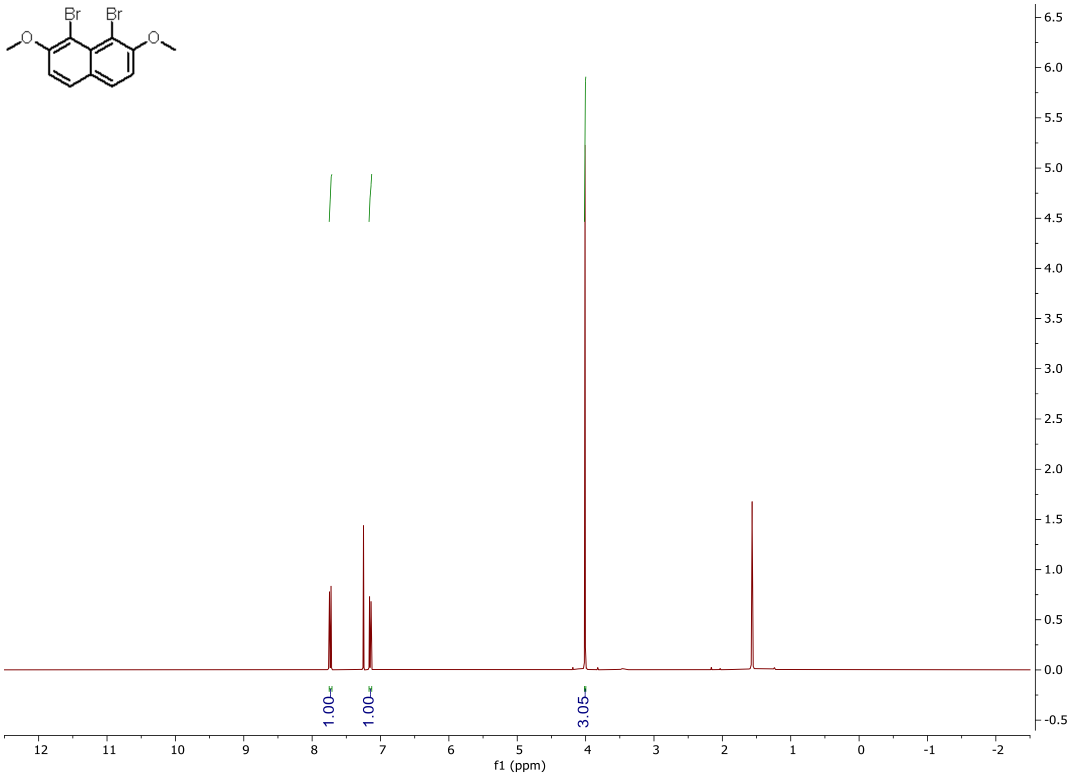

Figure S4. ^{1}H NMR of compound B

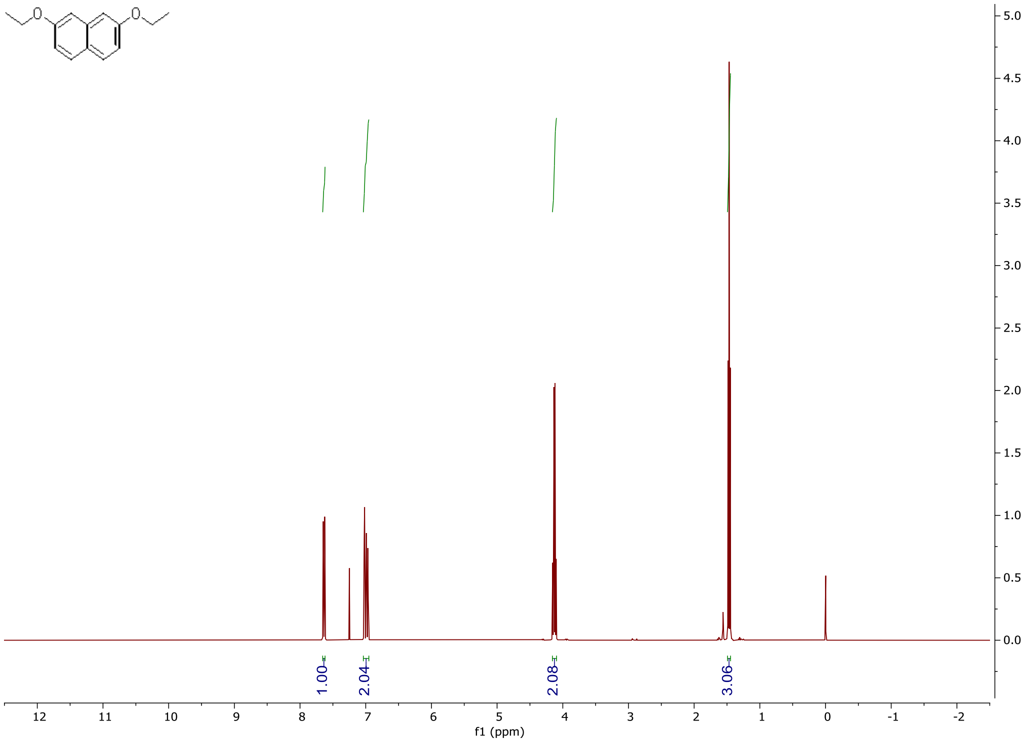

Figure S5. ^{1}H NMR of compound 4

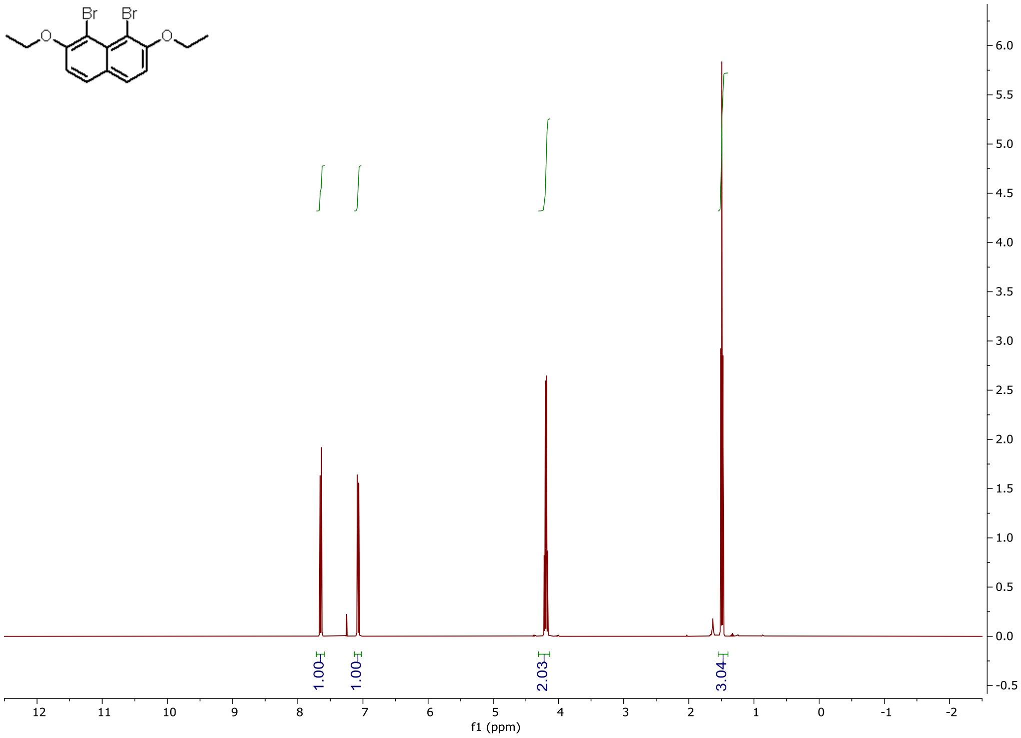

Figure S6. ^{1}H NMR of compound C

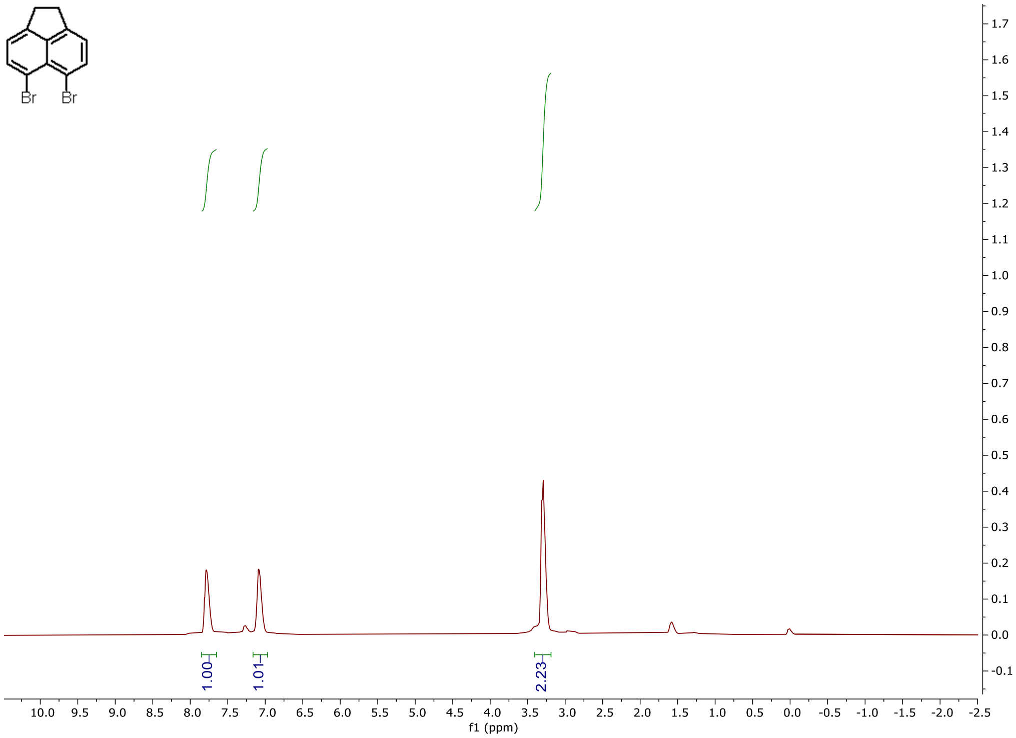

Figure S7. ^{1}H NMR of compound D

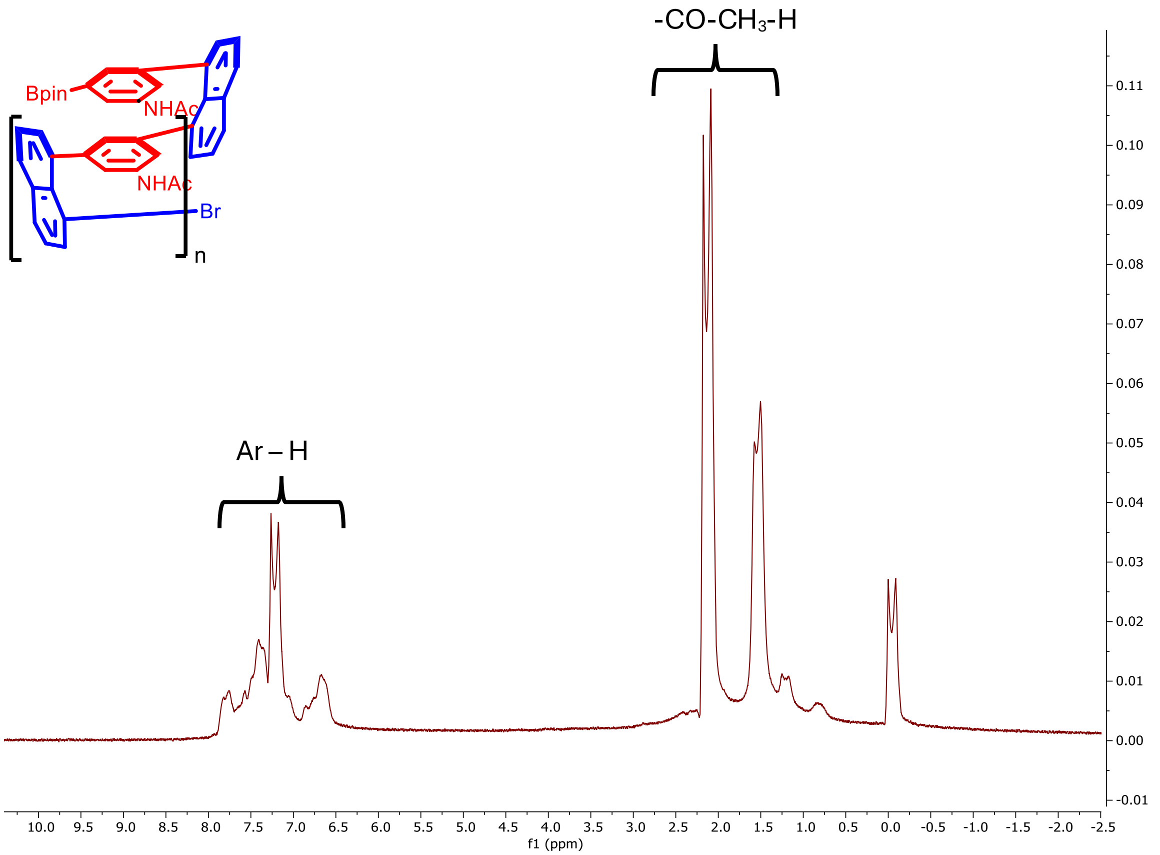

Figure S8. 1H NMR spectrum of 1A

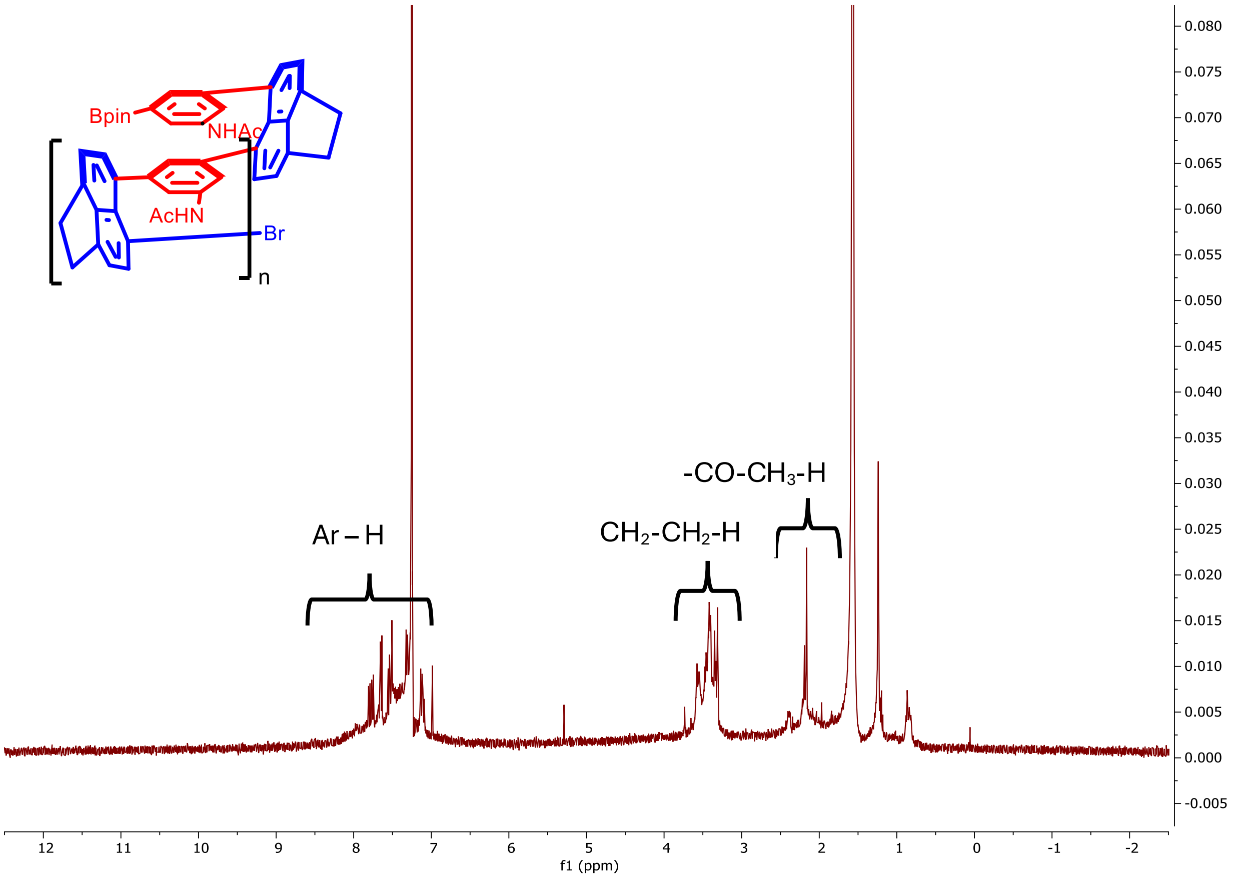

Figure S9. 1H NMR spectrum of 1B

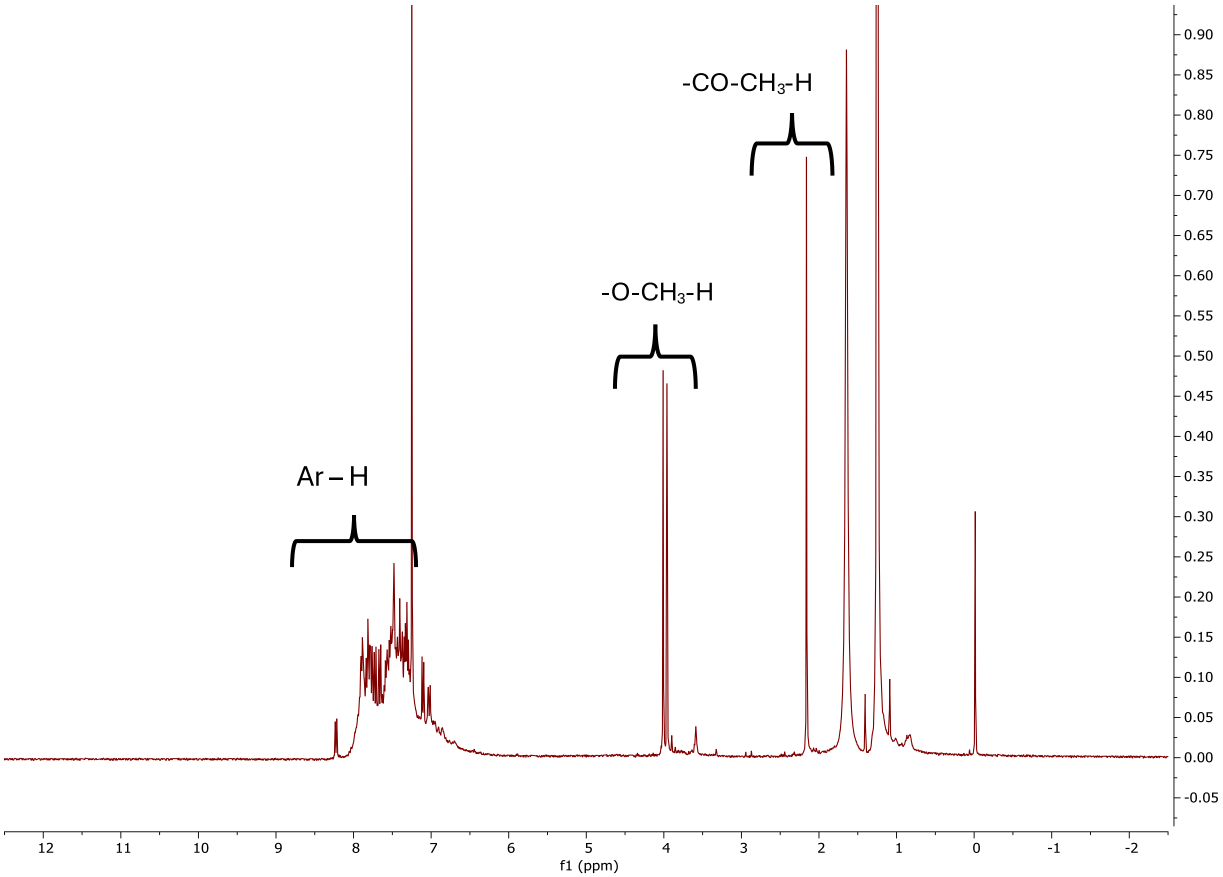

Figure S10. 1H NMR spectrum of 1C

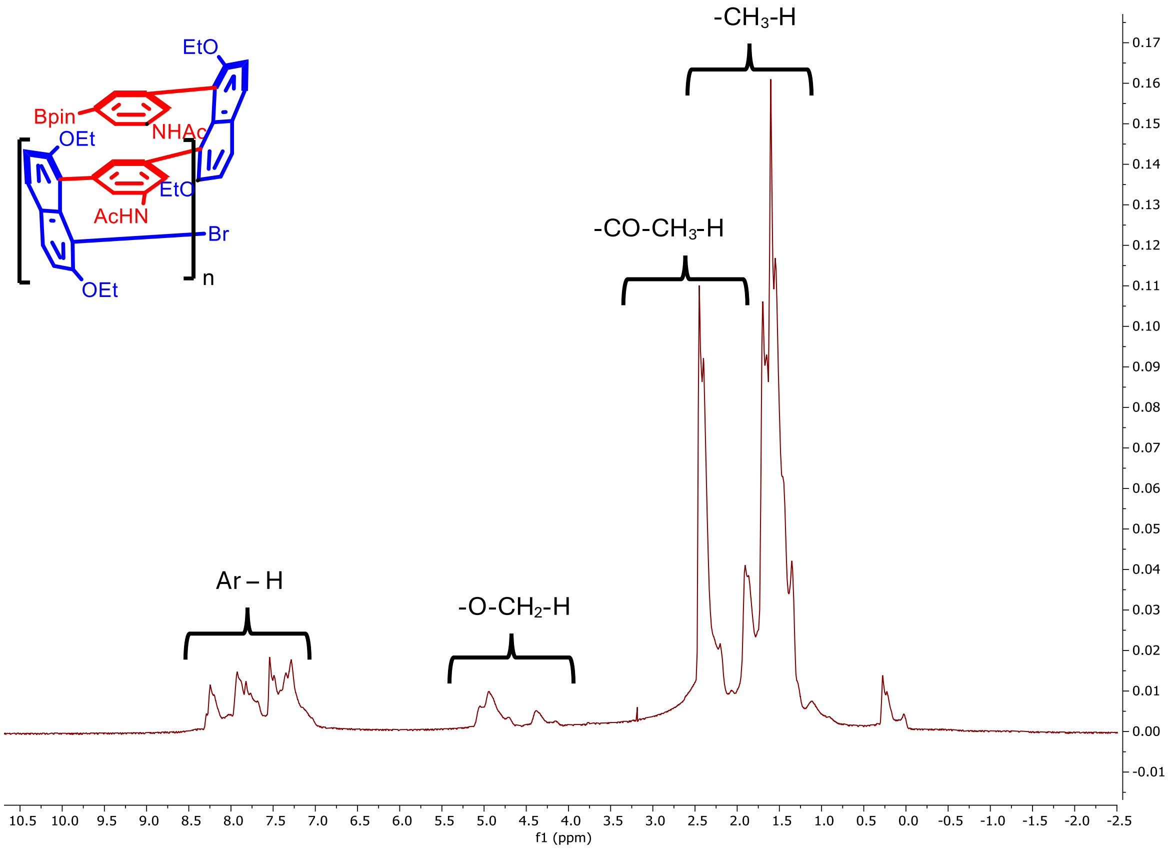

Figure S11. 1H NMR spectrum of 1D

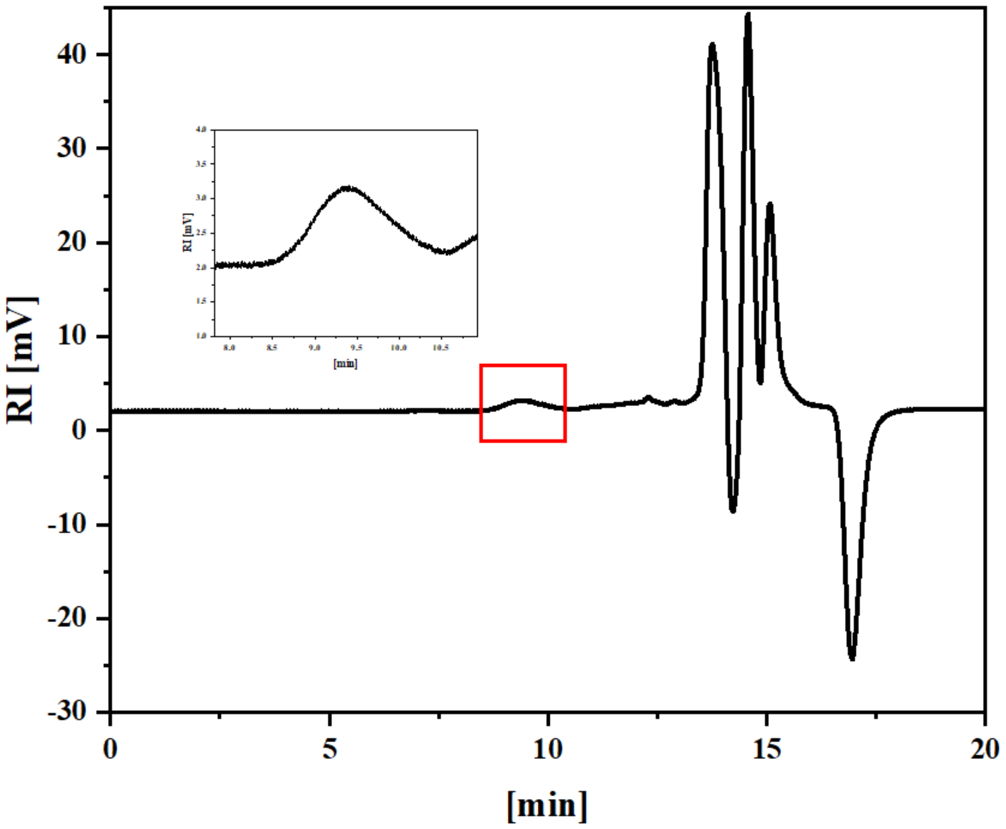

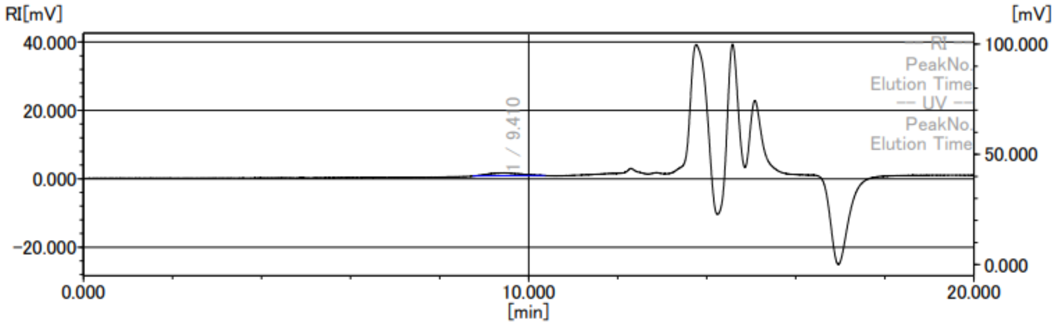

<Result of Molecular Weight Calculation>(RI)
Peak1Base Peak

	[min]	[mV]	[mol]
Peak Start	8.750	0.779	204203
Peak Top	9.410	1.729	81585
Peak End	10.348	0.937	22137
Height[mV]			0.885
Area[mV s]			43.698
Area%[%]			100.000
[Eta]			82582.77222

Mn	68016
Mw	82583
Mz	97698
Mz+1	111697
Mv	82583
Mp	81585
Mz/Mw	1.183
Mw/Mn	1.214
Mz+1/Mw	1.353

Figure S12. GPC data of 1A. The inset shows the zoomed in rectangular area

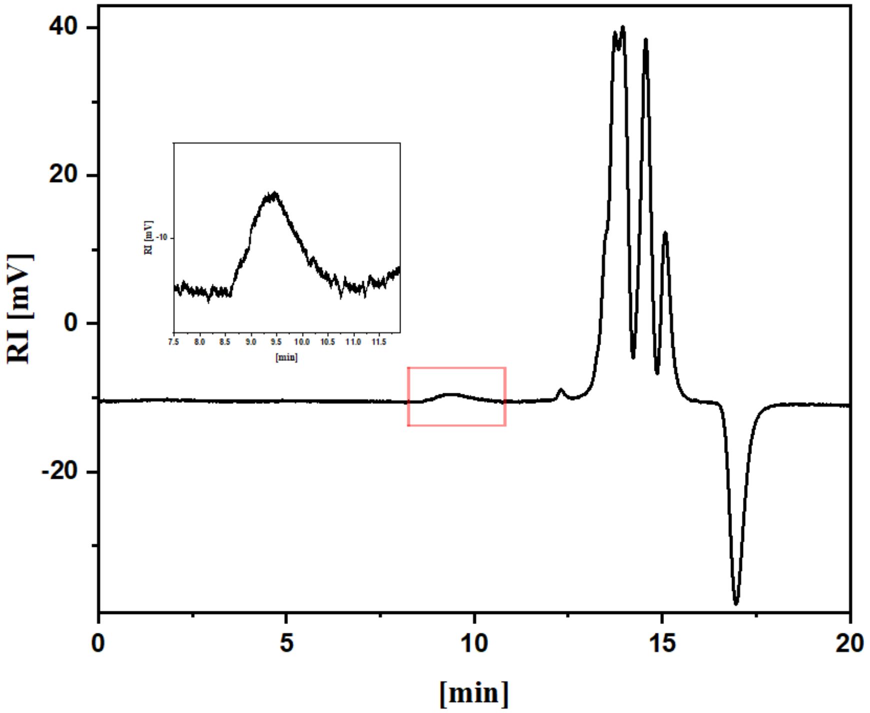

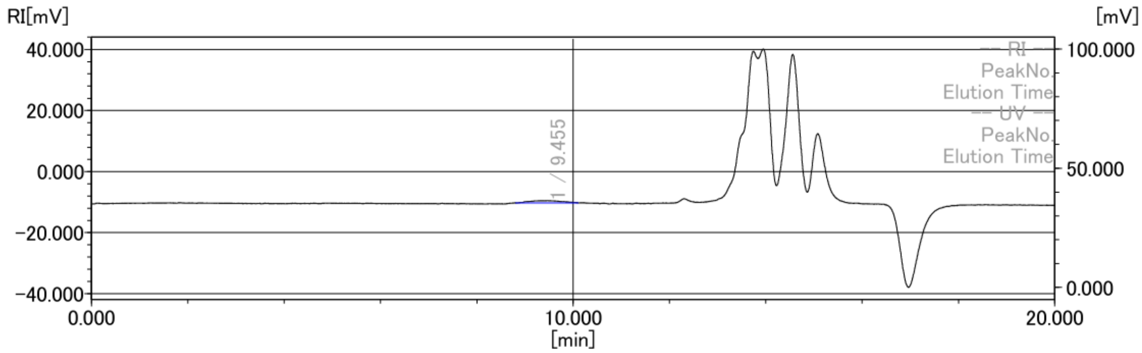

<Result of Molecular Weight Calculation>(RI)
Peak1Base Peak

	[min]	[mV]	[mol]
Peak Start	8.797	−10.277	191376
Peak Top	9.455	−9.508	76638
Peak End	10.110	−10.255	30832
Height[mV]			0.758
Area[mV s]			33.046
Area%[%]			100.000
[Eta]			84381.21086

Mn	72803
Mw	84381
Mz	96336
Mz+1	107547
Mv	84381
Mp	76638
Mz/Mw	1.142
Mw/Mn	1.159
Mz+1/Mw	1.275

Figure S13. GPC data of 1B. The inset shows the zoomed in rectangular area

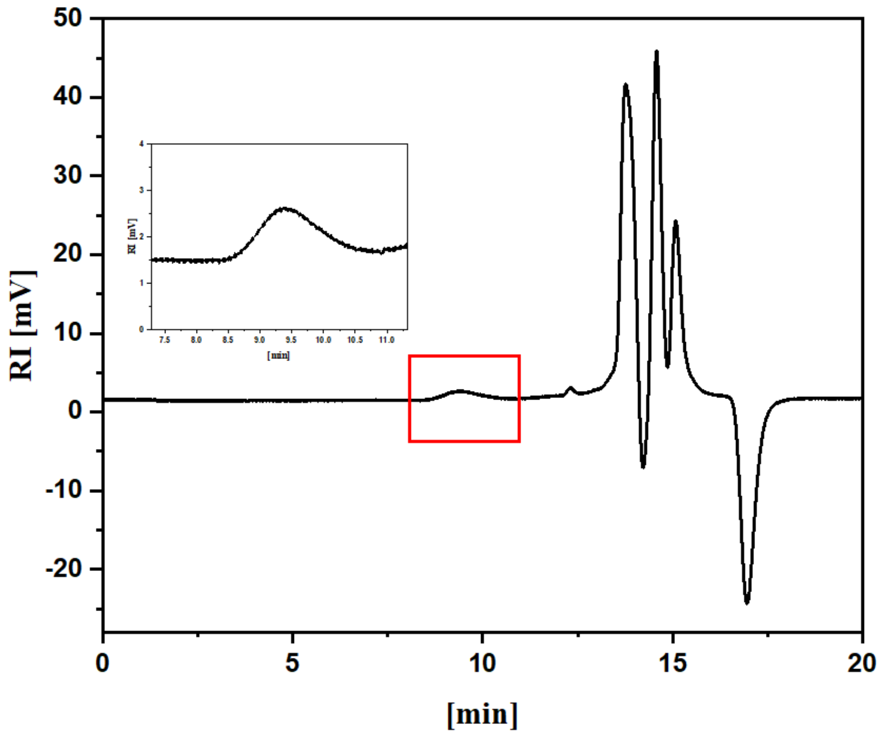

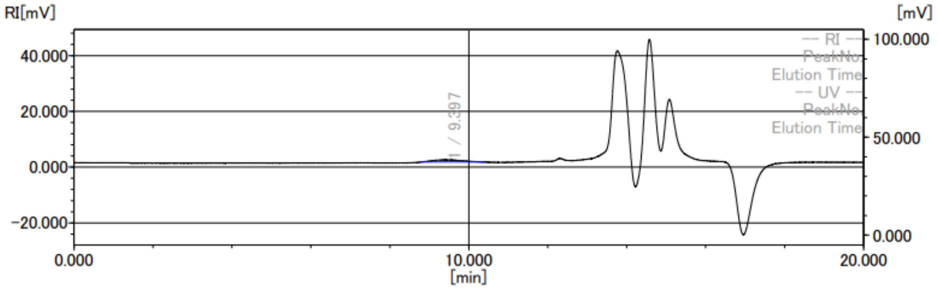

<Result of Molecular Weight Calculation>(RI)

Peak1Base Peak

	[min]	[mV]	[mol]		
Peak Start	8.765	1.801	199989	Mn	65917
Peak Top	9.397	2.655	83111	Mw	80674
Peak End	10.363	1.806	21680	Mz	95758
				Mz+1	109528
				Mv	80674
Height[mV]			0.852	Mp	83112
Area[mV s]			42.539	Mz/Mw	1.187
Area%[%]			100.000	Mw/Mn	1.224
[Eta]			80673.88742	Mz+1/Mw	1.358

Figure S14. GPC data of 1C. The inset shows the zoomed in rectangular area

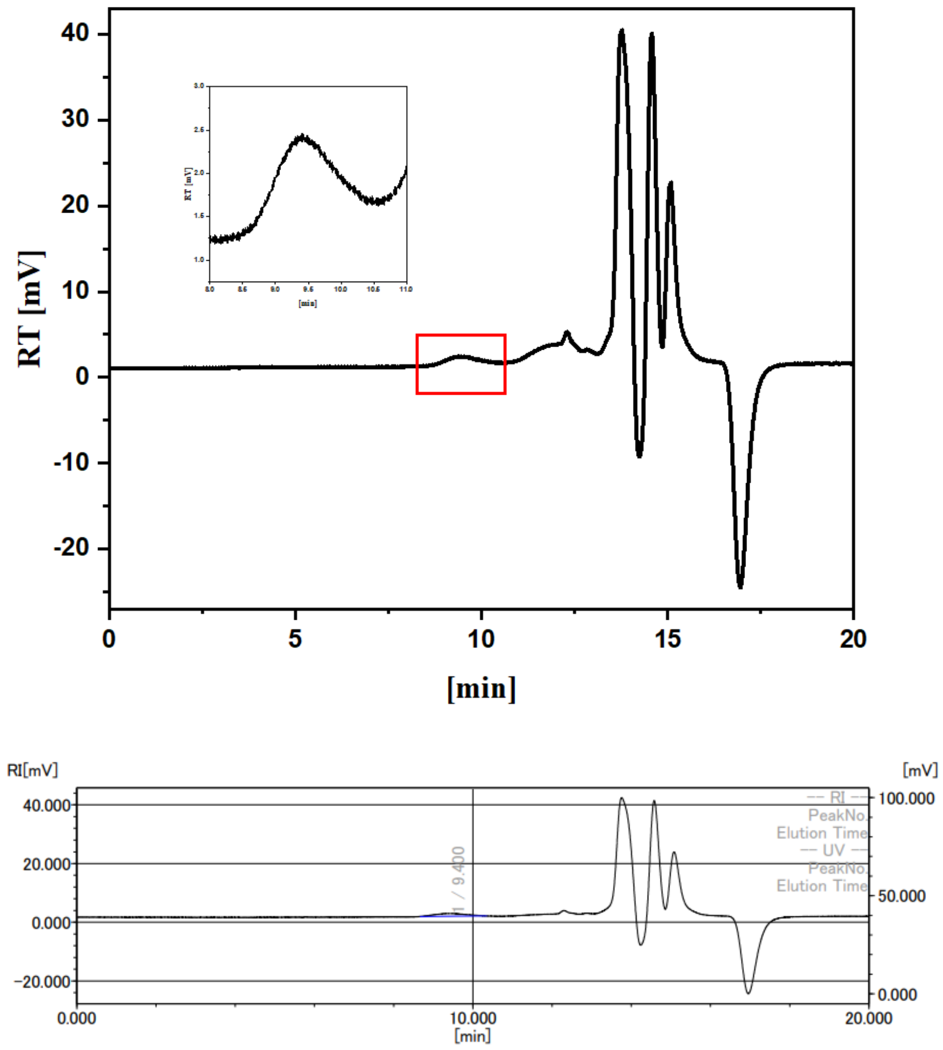

<Result of Molecular Weight Calculation>(RI)

Peak1Base Peak

	[min]	[mV]	[mol]		
Peak Start	8.672	1.966	227695	Mn	69165
Peak Top	9.400	3.008	82727	Mw	85674
Peak End	10.332	2.148	22656	Mz	103264
				Mz+1	119886
				Mv	85674
Height[mV]			0.962	Mp	82727
Area[mV s]			49.935	Mz/Mw	1.205
Area%[%]			100.000	Mw/Mn	1.239
[Eta]			85674.34090	Mz+1/Mw	1.399

Figure S15. GPC data of 1D. The inset shows the zoomed in rectangular area
